# A methodology to evaluate the optimal insulation thickness for heating and cooling needs in different climatic zones for buildings made of reinforced concrete with cavity walls

**DOI:** 10.1016/j.heliyon.2024.e30653

**Published:** 2024-05-06

**Authors:** D. Borelli, A. Cavalletti, P. Cavalletti, J. Peshku, L.A. Tagliafico

**Affiliations:** Department of Mechanical, Energy, Management and Transport Engineering, Division of Thermal Energy and Environmental Conditioning, University of Genoa, Via all’Opera Pia 15/A, 16145, Genoa, Italy

**Keywords:** Heating energy needs, Cooling energy needs, Annual energy convenience, Optimal insulation thickness

## Abstract

The study aims to define a methodology to evaluate the optimal insulation thickness with reference to the annual energy balance, including both heating and cooling seasons, for different climatic zones, based on three real case studies. The reduction of the heating needs due to the insulation of walls in places with cold climates is a consolidated concept. However, the effects of a high level of insulation of the envelope on the cooling needs has not yet been deeply analysed.

The heating and cooling needs have been computed according to UNI/TS 11300 Italian standard by means of a commercial numerical model, varying the sizes of the buildings, the climatic zone, and the level of insulation. All the simulations concern buildings made of reinforced concrete frames with cavity walls, because of their wide diffusion as typical building technology of post war constructions.

The work highlights the negative impact of the coating on the cooling needs, especially in the climatic zones with lower degree days. In almost all the climatic zones, the cooling need decreases its peaks during the hottest months (e.g., July or August), while the monthly load increases in the mild months (e.g., May, September) due to the additional thermal load which is no longer dispersed by means of the envelope. In conclusion, a range of optimal thicknesses for insulation for the different Italian climatic zones has been identified based on the total energy need of the building (cooling and heating).

## Highlights

• Buildings made of reinforced concrete frames with cavity walls were analysed.
• Heating and cooling needs were computed according to UNI/TS 11300 Italian standard.
• Buildings size, climatic zone, and level of insulation were varied.
• The negative impact of the coating on the cooling needs is highlighted.
• A general methodology to evaluate the optimal insulation thickness is described.

## 1 Introduction

Nowadays, energy consumption for domestic heating applications is one of the main factors causing global warming and climate change. In Italy residential and services sectors represented 43.5 % of total final energy consumption in 2018. In particular, the residential sector required about 34 Mtoe, with a yearly increase of about 1 % since 2000, due to two factors: more dwellings (4.6 Mtoe) and an increase in the level of comfort (4.5 Mtoe). To be more specific, space heating accounted for 69 % of the total consumption, followed by electrical appliances (13 %), water heating (11 %), cooking (6 %) and air-cooling (1 %.) [1].

For these reasons, the NECP (National Energy and Climate Plan) set different national targets to be achieved within 2030 for energy efficiency, renewable energy sources and reduction of CO_2_ emissions, energy security, development and sustainable mobility [2].

These goals were implemented by the Italian Government with specific rules concerning the reduction of energy consumption and introduction of renewable energies, for instance by means of the Italian Ministerial Decree on Minimum Requirements [3]. Moreover, the implementation of a series of dedicated fiscal incentives such as the so-called “Ecobonus” [4] and subsequent amendments, and, most recently, “Superbonus [5]”, stimulated the refurbishment of the Italian building stock, to improve the energy efficiency [6]. These laws and incentives are mainly focused on very strict transmittance limits for the building envelope, requiring the installation of insulation with relevant thickness (between 12 and 16 cm of an insulator with a conductivity of about 0.025 W/mK) even in locations with mild climate [3]**.** In fact, the higher the thermal resistance, the higher the energy saving, especially during the heating season.

Indeed, when the regulations above mentioned came into force, the heating need was dominant with respect to the cooling one. This issue is reflected by the carried-out research [[7], [8], [9], [10]], and it is due to the absence of widespread cooling systems at the time the laws came into force. For instance, it is estimated that the demand for air conditioners in Italy has almost doubled in ten years (from 2012 to 2021) [11,12].

In fact, the high initial cost, the unitary prices of electricity and the negligible cooling loads made the cooling systems an optional, leaving it to specific applications such as hospitals.

So, the energy saving strategy adopted in the past years was concerned with the insulation of the envelope, regardless of its effects on the building energy need out of the heating season [[13], [14], [15], [16]].

However, the diffusion of cooling systems increased dramatically during the past years. In 2021, the Italian National Institute of Statistics (ISTAT) estimated that about half of the Italian families had air conditioning or other cooling systems installed [11,12]. So, the need for a more complete analysis about the annual energy needs has become compulsory, accounting for both the initial costs and the energy savings. In particular, the energy savings are not directly proportional to the increase in the thickness, while the initial cost does [17]. In addition, the enhancement of the saved energy can lead to even relevant shifts in the optimal range of insulation thickness, especially considering the current economic and political situation.

In the studies available in literature, most of optimization analyses carried out during the 70's-90's are focused on the heating needs of buildings mainly located in Central and Northern Europe where the heating need is dominant if compared with the cooling one. The state of art of this topic is quite advanced since many ideas developed within scientific papers were implemented and followed by practical institutional guidelines [6,[18], [19], [20], [21], [22]]**.**

On the other hand, very few studies consider both cooling and heating loads and even fewer at milder locations, such as the Southern part of Europe or even hotter climates. In Ref. [23] an optimization model estimates the insulation thickness with reference to heating and cooling loads accounting for the Degree Days (DD) of the location, the energy conversion procedures and the price of primary energy (i.e. electricity or gas).

Another set of studies involved the insulation of roofs in southern locations, showing that solar reflectance acquires importance while the insulation loses relevance, with an optimal value reached at about 3 cm to optimise both cooling and heating loads (e.g. in Tunisia [24,25]).

A very similar result was found in a study concerning perimetral walls at different Turkish cities, at comparable latitudes [26], where the optimal value was between 3 and 4 cm of insulators with about 0.03 W/mK conductivity. On the other hand [27], finds similar but increased results with comparable boundary conditions (about 5–7 cm with 0.03 W/mK by means of a finite differences numerical model). Going to more Southern regions, such as in Qatar, the optimal insulation even decreases up to 1–2 cm of insulation with 0.03 W/mK [28].

Similar simulations were run also in Italy [29], showing that the building must not be over insulated and that for the Central-Southern Italian cities, the optimal insulation thickness for both heating and cooling loads might be lower than the minimum established by the law.

Concerning the colder regions, such as Estonia [30], the optimal insulation arises up to 15 cm of an average insulator with 0.03 W/mK conductivity. Another very interesting study carried out in China [31] identifies an optimal range between 5 and 18 cm of insulation with a conductivity of 0.03 W/mK, according to the climatic Chinese region. In particular, the wide interval of optimal insulation thicknesses is due to the high variability of the climate in China, starting from the coldest regions in the North, the cold winters and hot summers in the middle and the almost temperate/tropical climate in the South. Clearly, the highest values of thermal resistance are required where the heating load is dominant and vice versa, while the central regions require average values (e.g., about 10 cm) for optimal insulation since the heating and cooling needs become comparable. In Ref. [32], the economic and ecological optimal installation thickness was determined according to different climatic zones of Poland, different kinds of wall stratigraphy and heating sources and varying the type of the insulation material. For the residential apartment building sector of Lithuania, after the implementation of an investment in the building envelope covered initially 85 % by the investors, 15 % by the state with the obligation of the owners to pay the bill for heating (before the investment), a reduction of the energy need for heating 40–50 % and a reduction of the heating season was observed with a payback period of 12–15 years [33].

Then D'Agostino et al. [34] studied the interaction between variable thicknesses of insulation and increasing internal thermal loads at different locations. Both from the economic and energy sides, the result shows that an excessive level of insulation has to be avoided to reach an effective energy saving over the year. In addition, the adopted approaches can either work under simplified, steady state conditions (as in the case of [23] based on the DD or [35], or [36]) or consider the dynamic behaviour of the elements, as in Refs. [37,38] or in Ref. [39] where three typical Italian walls are studied with different levels of insulation. The transient simulations highlight the over-heating in excessively insulated walls, especially with high superficial mass, due to the higher thermal inertia.

All the cited works identify the most relevant variables of the optimum insulation thickness: the type of building and its thermal characteristics, the considered building element (e.g., roof, perimetral wall etc), orientation, climatic conditions, type and unitary prices of the energy sources. In every study, the optimal thickness never exceeds given thresholds since the material cost increases without a comparable benefit in the energy saving [[40], [41], [42], [43]].

The present work studies the effects of insulation on the heating and cooling loads for three different buildings of increasing dimension, located at different climatic zones, using a steady state approach given by UNI/TS 11300-1:2014 and UNI/TS 11300-3:2010. In the following section ‘Materials and Method’ the methodology for the calculation of the energy needs and the criterion for the definition of the optimal insulation thickness are presented.

## 2 Materials and methods

The methodology used to calculate the energy need for heating and cooling follows the approach described in the Italian national standards UNI/TS 11300-1:2014 - Energy performance of buildings - Part 1: Evaluation of energy need for space heating and cooling [44] and UNI/TS 11300-3:2010 - Energy performance of buildings - Part 3: Evaluation of primary energy and system efficiencies for space cooling [45] for the calculation of primary energy needs for summer air conditioning.

The approach proposed in the above recalled Technical Specifications performs an energy balance under steady state regime to estimate the heating (1) and cooling (2) energy needs:

(1) $$Q_{H,\text{nd}}=Q_{H,\text{ht}}\;-\;\eta_{H,\text{gn}}\;x\;Q_{\text{gn}}=\left(Q_{H,\text{tr}}+Q_{H,\text{ve}}\right)\;-\;\eta_{H,\text{gn}}\;x\;\left(Q_{\mathrm{int}}+Q_{\text{sol},w}\right)$$

(2) $$Q_{C,\text{nd}}=Q_{\text{gn}}\;-\;\eta_{C,\text{ls}}\;x\;Q_{C,\text{ht}}=\left(Q_{\mathrm{int}}+Q_{\text{sol},w}\right)\;–\;\eta_{C,\text{ls}}\;x\;\left(Q_{C,\text{tr}}+Q_{C,\text{ve}}\right)$$

where:

Q_H,nd_ and Q_C,nd_ are respectively the energy need for heating and cooling of the building. They represent the total energy need of the structure for climatization
Q_H,ht_ and Q_C,ht_ are the total thermal exchanges during the heating (H) and cooling (C) seasons;
Q_H,tr_ and Q_C,tr_ are the thermal exchanges due to transmission respectively during the heating and cooling season.
Q_H,ve_ and Q_C,ve_ are the thermal needs due to ventilation in the case of heating and cooling respectively;
Q_gn_ are the total thermal gains, composed by the internal (Q_int_) and the solar thermal gains through the transparent components of the envelope (Q_sol,w_).
η_H,gn_ is the utilization factor of the thermal gains;
η_C,ls_ is the utilization factor of the thermal losses.

Basing on the principle of superposition of the effects, both formulations perform a balance between two main terms: the former is represented by the thermal energy needs required to grant the set internal air temperature (I.e., 20 °C and 26 °C respectively for the heating and cooling season). The latter is part of free thermal gains/losses which contribute to the balance.

During the heating season, the energy losses through the envelope and due to ventilation constitute the thermal load. On the other hand, these two terms become free natural losses during the cooling season contributing to the heat dissipation.

Therefore, (1), (2) highlight the double role of insulation which reduces the amount of heat exchanged through the envelope causing both the reduction of the heating thermal load and the increase of the cooling need. In fact, a higher degree of insulation is optimal to minimise the thermal losses towards the external during the cold period, but it is not advisable during the cooling season when heat in excess has to be dissipated in other ways (I.e., using a heat pump).

The energy balances for the estimation of the heating/cooling need adopt a steady state approach, accounting for the transient effects by means of the utilization factor, either η _H,gn_ or η_C,ls_. It represents the amount of energy (gains for heating and losses for cooling) that really decreases the energy need. For instance, in summer part of the free thermal losses during the day might cause a subcooling below the set temperature with no effective reduction in the total cooling need.

The variations of the total energy need (I.e., both cooling and heating) were estimated basing on the recalled approach, by means of a commercial software that implements the approach of UNI/TS 11300.

So, the illustrated approach is based on monthly calculation of the thermal energy requirements, and it is widely adopted for different applications: from project calculation, assessment of the energy performance of buildings either in standard or specific climatic and operating conditions.

As concerns the present work, the described approach was used to evaluate the effects of increasing thicknesses of insulation on the total energy need (I.e., cooling and heating) on different buildings, varying their size and the climatic zone.

A numerical model of each considered case study was developed using Namirial Termo - Version 4.1.3 commercial software [46], performing calculations in conformity with UNI/TS 11300 part 1 and 2:2014, UNI/TS 11300-3:2010, UNI/TS 11300 part 4, 5, 6: 2016 and UNI EN 15193:2008, after carrying out a complete survey of each building to collect all the main characteristics regarding the envelope (both transparent and opaque components), the heating systems and the location. Then, the heating and cooling needs were computed for the Italian climatic zones B, C, D, E and F [47], considering for each one increasing thicknesses of insulation (from 0 cm up to 30 cm with 2 cm step). Climatic zone A has been neglected since it represents only two small municipalities all over the entire Italian territory. The insulation adopted in all the considered cases has a conductivity of 0.027 W/mK and it is applied to the external walls and to the roof of the buildings. All the thermal bridges were considered and updated to correctly estimate their contribution as the level of insulation increases. Their calculation was done by means of either CENED abacus or a two-dimensional finite elements numerical model developed with the THERM software [48]. In the case of numerical modelling, for each thermal bridge, the adopted mesh is the result of a convergence analysis performed to check the correct discretization of the nodes. The estimated value of transmittance per unit length was then implemented in the general model of the building. Increasing steps of 2 cm were used to identify the optimal insulation thickness for each case study, also based on a cost-benefit analysis in terms of energy costs. Actually, the current economic and political situation is causing unpredictable fluctuations of the gas, electricity and raw material prices, so the costs for the gas and the electricity could be different from the ones presented in this work [49]. Anyway, the results can still be considered acceptable since the increase of prices affects almost uniformly the studied scenarios. In this study the range of optimal insulation thickness is associated to a reduction of the annual energy cost lower than 2 %. In addition, the optimal range of insulating thicknesses will be compared to the current regulatory limits, which mainly consider the heating needs instead of the total ones (i.e., both cooling and heating). In fact, maximum transmittances (U_max_) for new/refurbished envelopes came into force in 2015 [3] and they have been recently revised [50] as shown in Table 1. More in general, these values are aligned with the ones adopted in other European countries for new/refurbished components and with the newly introduced European Regulation 2021/2139 [51] came into force on January 1^st^, 2022. Such Regulation aims to introduce the same set of transmittances for every EU country to establish if an activity contributes to climate change mitigation and causes no harm to environmental objectives.

**Table 1:** Maximum mandatory transmittances for roofs and vertical walls in energy efficiency refurbishments/new buildings.

Climatic zone	HDD	Vertical opaque walls		Roofs (horizontal or inclined)	
		U_max_ [W/m^2^K] – Minimum requirements [3] (including thermal bridges)	U_max_ [W/m^2^K] – Decree 06/08/2020 [50] (without thermal bridges)	U_max_ [W/m^2^K] – Minimum requirements [3] (including thermal bridges)	U_max_ [W/m^2^K] – Decree 06/08/2020 [50] (without thermal bridges)
A	0–600	0.54	0.38	0.32	0.27
B	601–900	0.41	0.38	0.32	0.27
C	901–1400	0.34	0.30	0.32	0.27
D	1401–2100	0.29	0.26	0.26	0.22
E	2101–3000	0.27	0.23	0.24	0.20
F	>3001	0.26	0.22	0.23	0.19

Actually, the two limits in Table 1 provide complementary information since one accounts for the thermal bridges, while the other is strictly related just to the stratigraphy of the elements. The introduction of a double limit is meant to grant both a proper level of envelope insulation and the correction of the thermal bridges as well.

### 2.1 Description of the case studies

Very different building typologies can be identified in Italy and more in general in Europe, according to the construction techniques, materials and total height. In general, the energy efficiency of the existing buildings is poor especially in territories characterised by mild climates. The present study is focused on the buildings made of reinforced concrete with cavity walls, which mostly affects the energy consumption according to data collected by the Italian National Institute of Statistics (ISTAT) [52].

**Fig. 1:**
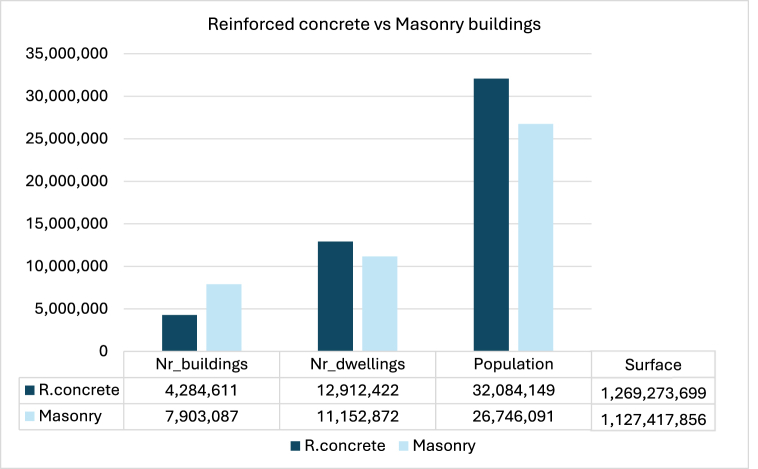
The building stock in Italy [52].

Fig. 1 shows that reinforced concrete with cavity walls and masonry buildings are the two most spread building typologies over the Italian territory. Although the former is about half of the latter, more population and dwellings can be found in the reinforced concrete buildings. Indeed, this kind of construction is very common especially after World War II and it allows an increase in the number of storeys, to accommodate more families. For this reason, the investigation was carried out considering the reinforced concrete with cavity walls buildings.

**Fig. 2:**
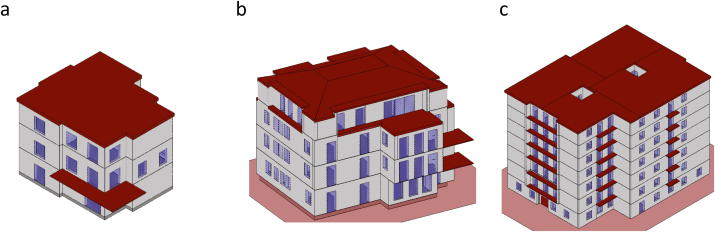
3D model of the single-family building (a), semi-detached building (b) and a multistorey apartment building (c) used for the study.

The analysed buildings are real case studies, all used for residential applications with increasing sizes. In particular, in this study are considered a single-family building (Fig. 2a), a semidetached four-family building (Fig. 2b) and an apartment building of seven floors (Fig. 2c) made of reinforced concrete and cavity walls.

**Fig. 3:**
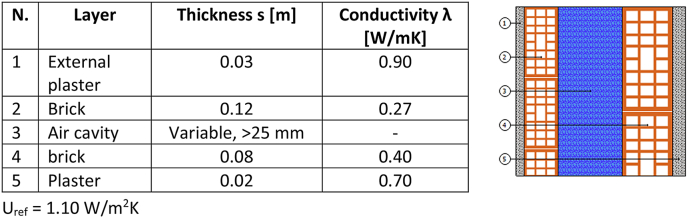
Most recurrent stratigraphy of the external wall and the expected average transmittance.

All the case studies were subject to an accurate survey, to assess the thermal properties of their envelopes. Actually, all the buildings date back to the same construction period and therefore they present very similar characteristics. The two main reference components (perimeter wall and roof) are reported below, while all the other stratigraphies were omitted for the sake of brevity. Fig. 3 shows the reference stratigraphy of the cavity wall, one of the most recurrent opaque elements: two layers of bricks are separated by an air cavity with variable thickness from one building to the other. Anyway, the cavity is usually larger than 25 mm and therefore the thermal resistance is considered constant for thicknesses higher than 25 mm, as prescribed in the standard UNI 6946:2018 [53]. As a consequence, any variation in the perimetral wall thickness is associated to different sizes of the air cavities (but still higher than 25 mm), so from an energy point of view the thermal resistance can be assumed unchanged among the different cases. All thermophysical parameters have been chosen using the original design, the available information and the data reported in the UNI/TR 11552:2014 [54] standard.

**Fig. 4:**
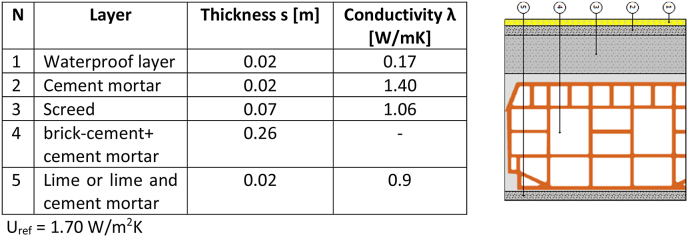
Most recurrent stratigraphy of the roof, expected average transmittance.

On the other hand, the roof is usually made of brick-cement with additional layers of screed and tiles or waterproof layer as in the case presented in Fig. 4.

The range of minimum thickness to be compliant to the legislative limits (Table 1) by means of an insulator with conductivity λ = 0.027 W/mK clearly depends on the number and the extension of the thermal bridges and it is briefly identified in Table 2 assuming as initial values the transmittances reported in Fig. 3, Fig. 4. The table aims to identify the range of required thickness for the insulating layer, according to the main opaque envelope and the climatic zone. This minimum operative range will be then compared to the optimal one reported in the results.

**Table 2:** Average range of thickness required to respect the maximum transmittances for the reference opaque vertical wall (Fig. 3) and for the reference roof (horizontal or inclined, Fig. 4).

Climatic zone	Average range of thickness to respect the limits (Table 1), assumed 0.027 W/mK as conductivity of the insulator	
	Opaque vertical wall [cm]	Roof (inclined or horizontal) [cm]
A	7–8	10–12
B	7–8	10–12
C	9–10	10–12
D	10–12	13–15
E	11–14	14–17
F	12–14	15–18

All the considered buildings are provided with a centralized gas burner system, usually interfaced with radiators, while no cooling is present. In fact, natural gas is the energy source most widespread (81.9 % during 2021) while most of the buildings are not provided with centralised cooling systems, but only single/double room units which have not been designed to cover the cooling load of the building/flat [55].

Table 3 resumes the main information about the external climate of the five Italian cities chosen to represent the different climatic zones.

**Table 3:** Information about the cities considered in the simulations.

Zone	City	Medium seasonal temperature [°C]	Heating Degree Days	Design external temperature [°C]	Annual average temperature [°C]
Zone B	Palermo	12.3	751	5	18.8
Zone C	Imperia	10.2	1201	2	15.8
Zone D	Genoa	11.2	1435	0	16.5
Zone E	Milan	7.7	2404	−5	14.3
Zone F	Trento	5.8	3001	−12	12.5

More in detail, the following paragraphs resume the main characteristics of the three chosen buildings (Fig. 2). No relevant external obstructions except for the ones due to the geometry (e.g., balconies) are considered in this study, in order to make the different buildings comparable. Any obstruction would likely cause an increase in the heating needs and a decrease of the cooling ones since it reduces the solar gains. This issue shall be enquired in detail in future studies as reported in the conclusions. In all the presented cases, The variations in the thickness of the perimeter walls have been included as well, such as the part of wall below a window, which is usually thinner, or the wall towards the staircase.

*Case study 1 Single family building* (Fig. 2a)

- *Structuralcomponents:*
  ◦ *Opaque:* frame of reinforced concrete with 30 cm thick, not insulated cavity walls (Fig. 3); flat roof made of 30 cm brick-cement floor (Fig. 4).
  ◦ *Transparent:* PVC frames with double glass (1.3 W/m^2^K) covering about 7.6 % of the total surface.
- *Geometry:* Three-storey building with a useful heated surface is 218.8 m^2^ and the ratio between the dispersing surface S and heated volume V is S/V = 0.818.
- *Boundaries:* besides of the external, the ground floor is above an unheated space used as technical room and gym.

*Case study 2 Semi-detached building* (Fig. 2b)

- *Structural* *components:*
  ◦ *Opaque*: frame of reinforced concrete with 40 cm thick, not insulated cavity walls (Fig. 3, larger air cavity, no relevant change in the total transmittance of the wall), flat roof made of 30 cm brick-cement floor (Fig. 4).
  ◦ *Transparent*: metal frames with double glass (4.2 W/m^2^K) covering about 10.7 % of the total surface.
- *Geometry:* four-storey building with a useful heated surface is 1512.13 m^2^ and the ratio between the dispersing surface S and heated volume V is S/V = 0.504.
- *Boundaries:* The perimeter of the ground floor confines with a crawl space, while all building presents an internal, unheated staircase.

*Case study 3 Apartment building* (Fig. 2c)

- *Structural* *components:*
  ◦ **Opaque*:* frame of reinforced concrete with 30 cm thick, not insulated cavity walls (Fig. 3), flat roof made of 30 cm brick-cement floor (Fig. 4).
  ◦ o *Transparent:* metal frames with single glass (6 W/m^2^K) covering about 12 % of the total surface.
- *Geometry:* seven-storey building with a useful heated surface is 4686 m^2^ and the ratio between the dispersing surface S and heated volume V is S/V = 0.359.
- *Boundaries:* this building has two atria of 4 m × 3 m (see Fig. 2c), increasing the external dispersing surfaces. This geometric element becomes quite common in large buildings while it is usually absent in small and medium ones. The ground floor is in contact with other unheated spaces (e.g., garages).

## 3 Results of the case studies

The following results are related to the monthly energy needs (heating – positive values, cooling – negative values) for each of the three reference buildings, varying both the climatic zone and the thickness of the insulating coating as previously outlined. The design temperatures to be granted during the heating and cooling seasons are respectively 20 °C and 26 °C. Only the relevant thicknesses have been plotted to make the figures clearer. More in detail, the charts reported below refer to the following cases: absence of insulation, minimum thickness (i.e., 2 cm), insulation thickness within the range required to respect the legislative limits (i.e., variable according to the climatic zone as outlined in Table 2) and maximum considered thickness (i.e., 30 cm). This clearly represents a case limit, not corresponding to a real, feasible configuration. It is just meant to identify a minimum achievable energy need associated to a heavy coating.

### 3.1 Monthly energy needs

**Fig. 5:**
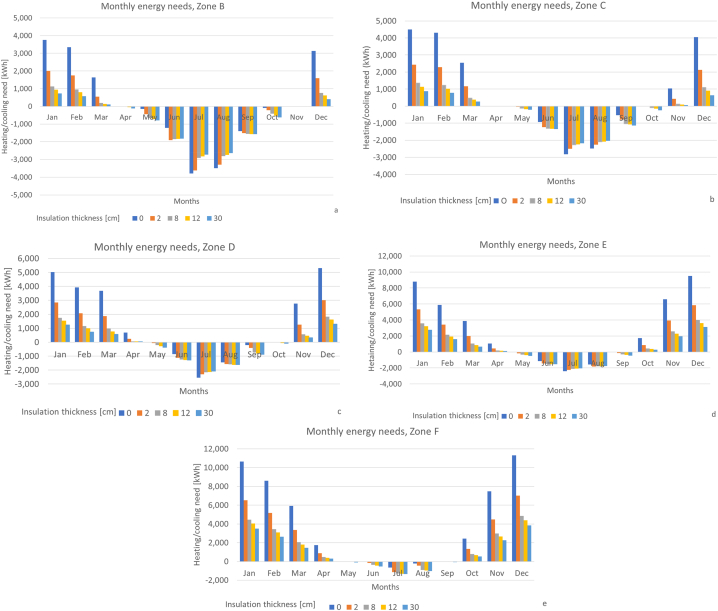
Monthly energy needs (heating – positive values, cooling – negative values) for a single-family building, varying the thickness of the insulator, at different climatic zones (a – climatic zone B, b – climatic zone C, c – climatic zone D, d – climatic zone E, e − climatic zone F).

Fig. 5 resumes the monthly energy needs for a single-family building as previously described. The results regarding the other case studies are reported in Annex 1.

The comparison of the charts in Fig. 5 can lead to the following observations:

*Heatingneeds:* independently from the climatic zones, it can be noticed that the heating needs dramatically drop as only 2 cm of insulation are added. Indeed, this thin coating at least halves the total heating needs for the single-family building, reaching a 60 % reduction in the climatic zone F. Even the step from 2 cm to 8 cm leads to relevant benefits on the heating needs with a further reduction of 23–27 %. These trends become more relevant as the climatic zone changes from B to E or F where the heating needs become more dominant. The following step (from 8 cm to 12 cm) decreases of only 4 % almost independently from the climatic zone. In the end the decrease of the heating needs corresponding to an insulator 30 cm thick is of 2–7% if compared to the previous intervention.

*Cooling needs:*

- as concerns the climatic zones from B to D, the peaks occurring during July and August show a small decrease (about 10 %) as 2 cm of insulation is installed in comparison to the not insulated scenario. The further increase of insulation determines the reduction of the cooling demand of only some percentage points. On the other hand, the cooling needs increase during the mild months (e.g., May or September) already with the installation of 2 cm thick insulation (about 20 %). Any further increase in the insulating thickness determines very small fluctuations (lower than 5 %). More in general, the addition of insulation causes a flattening of the peaks during the hottest months while an expansion of the cooling season can be observed. Considering the annual cooling needs, a global increase of about 8 % has been identified as a consequence of an insulation 2 cm thick. The other cases regarding 8, 12 and even 30 cm just determine limited increases (about 1–2%).
- Considering the climatic zones E and F, a coating 2 cm thick just determines an increase in the cooling needs during all the months, since the cooling demand of the not insulated case is already very small. As illustrated above, the other thicknesses (i.e., 8, 12, 30 cm) just cause a negligible increase (about 1–2%).

*Totalneeds:* In terms of the total energy needs (i.e., cooling and heating), an insulation of 2 cm decreases the total demand of about 25 % in climatic zone B, up to 40 % in climatic zone F. The following steps (from 8 cm to 12 cm) add a further decrease of 15–20 % in all climatic zones. In the end, the limit case of 30 cm leads to a very small decrease, about 2–7% according to the different climatic zone considered.

The same trends described above for heating, cooling and total demand can be found in the other case studies (i.e., semi-detached building, Figure A 1 and apartment building, Figure A 2) with the difference that the benefits of insulation during the heating season are reduced. The main reason is associated to the lower ratio between the dispersing surface S and heated volume V which reduces the impact of insulation on the energy need, as briefly shown in Table 4.

**Table 4:** Average reduction of the heating demand with an insulation 2 cm thick.

Case study	S/V [m^−1^]	Average percentage reduction of the heating demand [%]
Single family building	0.818	50
Semi-detached building	0.504	30
Apartment building	0.359	27

### 3.2 Optimal thickness for insulation

**Fig. 6:**
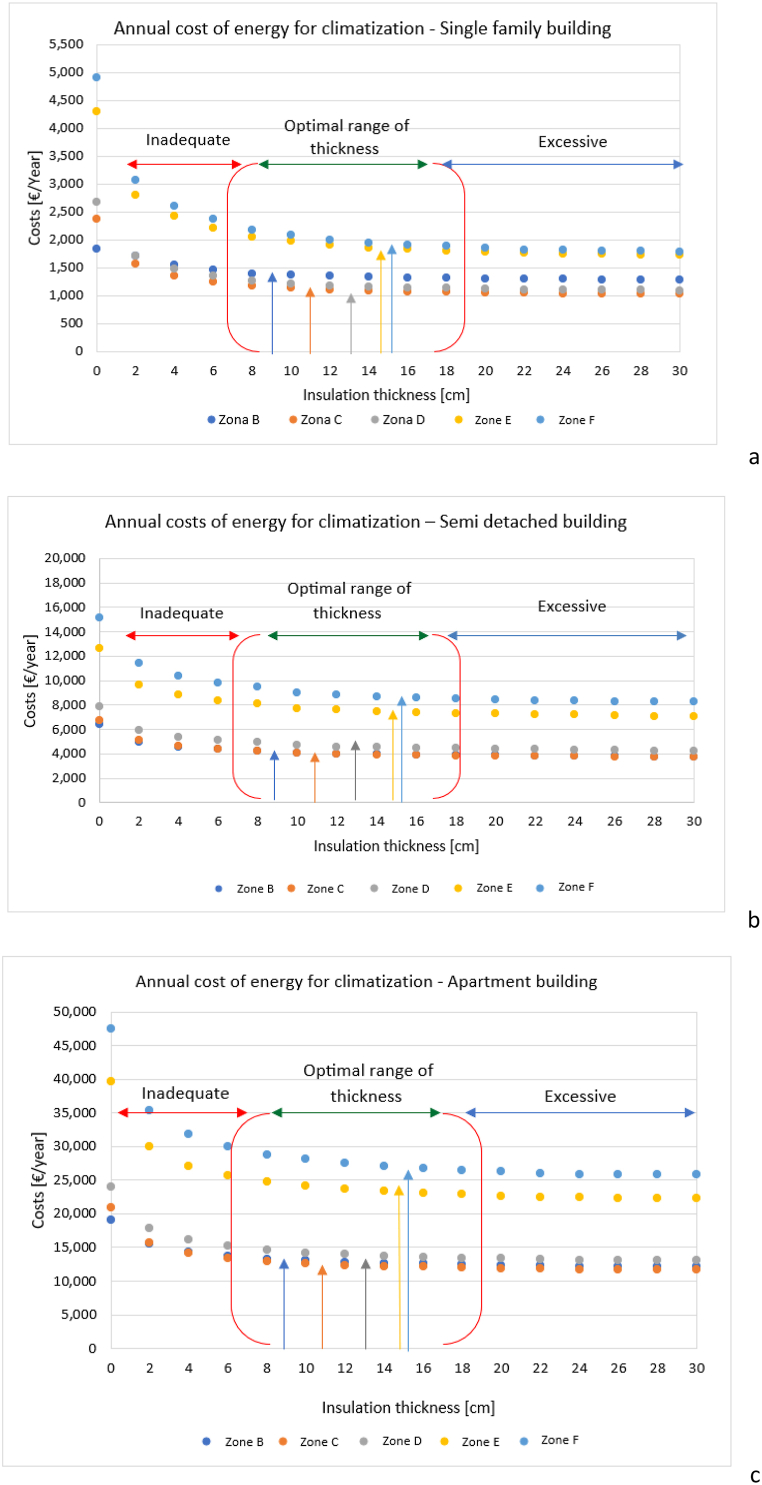
Optimal insulation thickness (shown with different coloured arrows) considering heating and cooling season for single family building – a; semi-detached building – b and apartment building – c.

The optimal insulating thickness is identified for the different case studies relating the energy reduction estimated in the previous section to the economic achievable savings. The presented analysis estimates the expense [€/year] referred to the annual total energy needs of the building (cooling + heating) considering a gas burner as standard heating plant (efficiency 0.8, unitary price for gas 0.1 €/kWh) and a heat pump-based technology for cooling (COP = 3, unitary price for electricity 0.32 €/kWh). The optimal value is then associated to the minimum thickness of insulation after which no further, relevant reduction of the annual cost is appreciated (i.e., lower than 2 %). Fig. 6 resumes the savings for the three case studies (a – single family, b – semi-detached, c – apartment building).

For every considered case, a dramatic reduction occurs in the very first thickness-steps, namely from 2 cm up to about 10 cm. For larger thicknesses, the trend rapidly becomes asymptotic, showing the lack of benefits associated to a heavy coating. Furthermore, the optimal insulation thickness shows a very weak sensitivity to the ratio between the dispersing surface (S) and the heated volume (V). On the other hand, the climatic zone does influence the optimal values, as also resumed in Table 5.

**Table 5:** Comparison between the optimal insulation thickness carried out int the study and the range of thickness mandatory to respect the limit transmittance.

		Optimal insulation from cost benefit analysis [cm]		
Climatic zone	Insulation range [cm] to respect the legislative limits assuming 0.027 W/mK - Table 2	Single family building (S/V = 0.818 1/m)	Semi-detached building (S/V = 0.504 1/m)	Apartment building (S/V = 0.359 1/m)
B	7–12	8–10	8–10	8–10
C	9–12	10–12	10–12	10–12
D	10–15	12–14	12–14	12–14
E	11–17	14–16	14–16	14–16
F	12–18	14–16	14–16	14–16

The table above also shows a that the range of thicknesses identified by means of the cost-benefit analysis is almost always contained into the one required to respect the maximum regulatory transmittances (see Table 2 for further information). This highlights the need to avoid the adoption of stricter limits for the thermal resistance, since no relevant improvements to the building energy efficiency would be achieved in spite of the side effects resulting from the installation of thicker coatings (e.g., increased cost, limited space on the facades, compatibility of insulation with traditional old materials also from an architectural point of view).

**Fig. 7:**
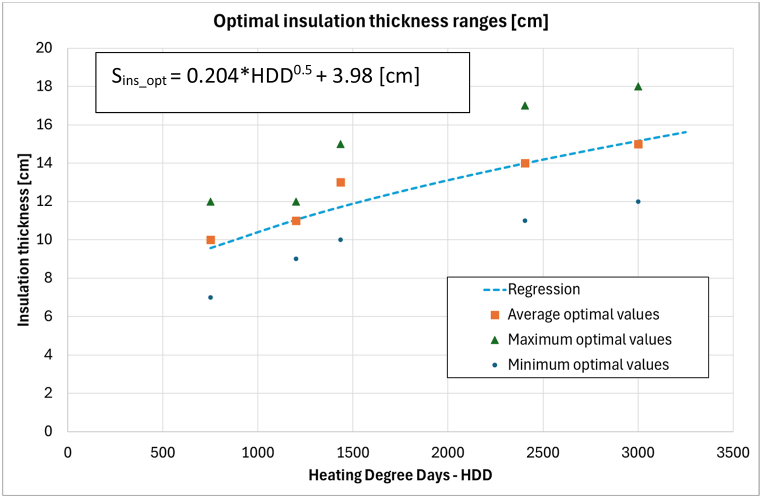
Optimal insulation thickness according to the heating degree days (HDD). Simulated values and the regression line.

To conclude, the results of the simulations have been plotted against the Degree Days for each city considered (Fig. 7), showing a good approximation with the correlation which is showed in the figure itself. This equation represents a very useful tool to preliminarily assess the optimal insulation thickness only according to the location.

## 4 Conclusions and future developments

The present work investigates the variation of both cooling and heating needs accounting for external insulating coating, basing on the approach described in the Italian national standard UNI/TS 11300.

Three kinds of buildings of increasing sizes (single-family, semi-detached building and apartment building) were studied in the five, most common Italian climatic zones (i.e., from B to F) with an external insulation of increasing thickness (from 0 cm up to 30 cm with 2 cm steps). The characteristics of the envelopes were chosen basing on a statistical analysis about the Italian residential existing building stock.

The work evaluates the variation of the total energy needs (cooling + heating) for a building, considering the insulations with increasing width. In addition, an optimal range is identified choosing the minimum thickness after which the reduction in the total energy needs (cooling + heating) is negligible (i.e., lower than 2 %).

In particular, a general decrease in the heating need is found for all the buildings in all climatic zones as thicker insulations are adopted. Actually, the highest reductions with respect to the not insulated case (about 30–50 %) are reached with just 2 cm of insulation, while the additional thicknesses determine even smaller reductions. As far as the cooling needs are considered, the insulation of the envelope causes an expansion of the cooling season, with a reduction of the single monthly peaks. Globally, the total cooling needs of insulated buildings increase of about 50 % as the Degree Days of the location increase (e.g., zone E or F), if compared with the not insulated case. Clearly, the cooling needs associated to climatic zones E or F are still about an order of magnitude smaller than the ones of the climatic zones with low Degree Days (i.e., B or C). In addition, the heating needs resulted dominant for all the considered buildings and climatic zones.

Besides of the side effects of the insulating coating on the cooling needs and the maximum attainable energy savings, the study identifies a range of optimal insulation thicknesses out of which the insulation is either poor (i.e., lower values) or excessive (i.e., higher values) without any effective reduction in the total energy needs and the cost benefit analysis. The carried-out simulations highlighted that while the energy savings associated to insulation depend on the S/V ratio (S – dispersing surface and V – heated volume), the optimal range of insulating thickness has a very low sensitivity to this parameter while it mainly depends on the climatic zone.

In addition, the optimal ranges fall within the regulatory insulating thicknesses required to respect the maximum limit transmittance. Stricter values of maximum allowable transmittance would result in no relevant savings in the energy needs of buildings, in front of the additional costs and the downsides associated to a thicker coating.

In conclusion, a correlation has been proposed to identify the optimal insulation thickness according to the Degree Days of the climatic zone, basing on the simulated data. The equation represents a very useful tool to preliminary estimate the optimal insulation and the methodology proposed can be easily adapted and replicated for other countries with different standards and climates. Indeed, the considered maximum transmittances are aligned with the ones adopted in the recent European Regulations. As far as the future developments are concerned, the analyses should include following topics:

- *Cost benefit analysis:* the optimal thickness for an insulating coating could be refined also considering the cost-benefit analysis associated to the initial installation cost. Actually, this kind of studies is very likely to become outdated fastly, in particular nowadays because of the steep fluctuations in the costs of the materials caused by the current fiscal incentives and policies.
- *Influence of obstructions:* the cooling needs show a strong dependance on the solar radiation and therefore different obstructions and shadings could play a not negligible role in the assessment of this parameter.
- *Extension of the analysis to other opaque stratigraphies:* for instance, in Italy there is still a relevant number of old buildings made of masonry walls and timber roof which should be accounted.
- *Inclusion of the climatic zone A:* even if preliminary conclusions can be drawn by extrapolation of the results for zone B, a more comprehensive study could be carried out to obtain real data.
- *Compatibility of the optimal insulation with the boundaries of existing buildings:* the range of thicknesses shown in the table above has to be checked also from the point of view of feasibility and compatibility with real cases. Most of buildings show critical features where even a coating 8 cm thick could not be applied, for instance due to the lack of space.
- *Refinement of the equation relating the optimal thickness for insulation to the Degree Days:* the equation can be better formulated by simulating more cities and eventually adding the dependance on other parameters such as the already mentioned obstructions.

## CRediT authorship contribution statement

**D. Borelli:** Investigation, Supervision, Validation, Writing – review & editing. **A. Cavalletti:** Investigation, Supervision, Validation, Visualization, Writing – original draft. **P. Cavalletti:** Resources, Supervision, Writing – review & editing, Conceptualization, Methodology. **J. Peshku:** Writing – original draft, Data curation, Formal analysis, Software, Visualization. **L.A. Tagliafico:** Supervision, Validation, Writing – review & editing.

## Declaration of competing interest

The authors declare that they have no known competing financial interests or personal relationships that could have appeared to influence the work reported in this paper.

## Figures and Tables

**Fig. A1:**
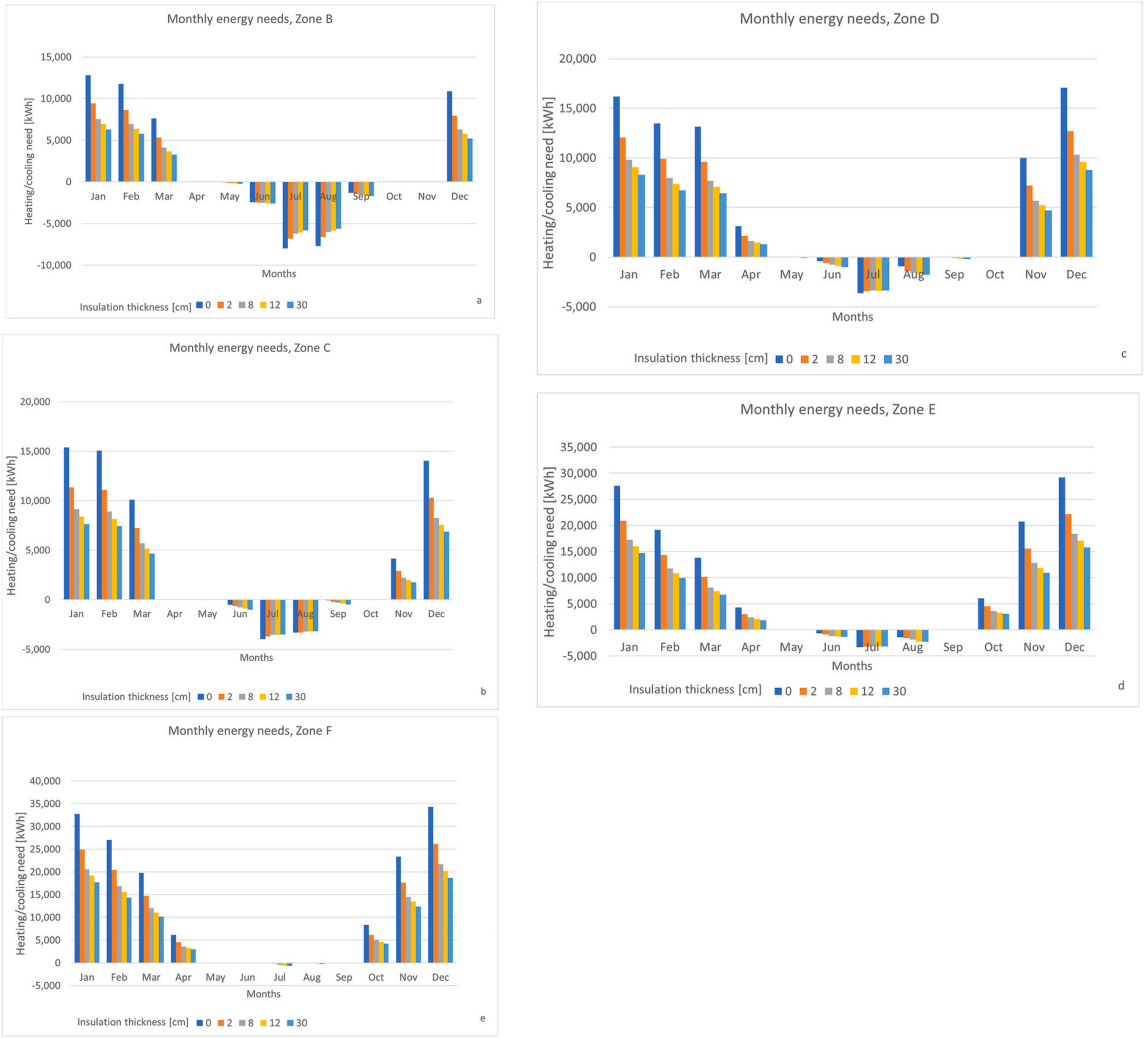
Monthly energy need (heating – positive values, cooling – negative values) for a semi-detached building, varying the thickness of the insulator, at different climatic zones (a – climatic zone B, b – climatic zone C, c – climatic zone D, d – climatic zone E, e − climatic zone F).

**Fig. A2:**
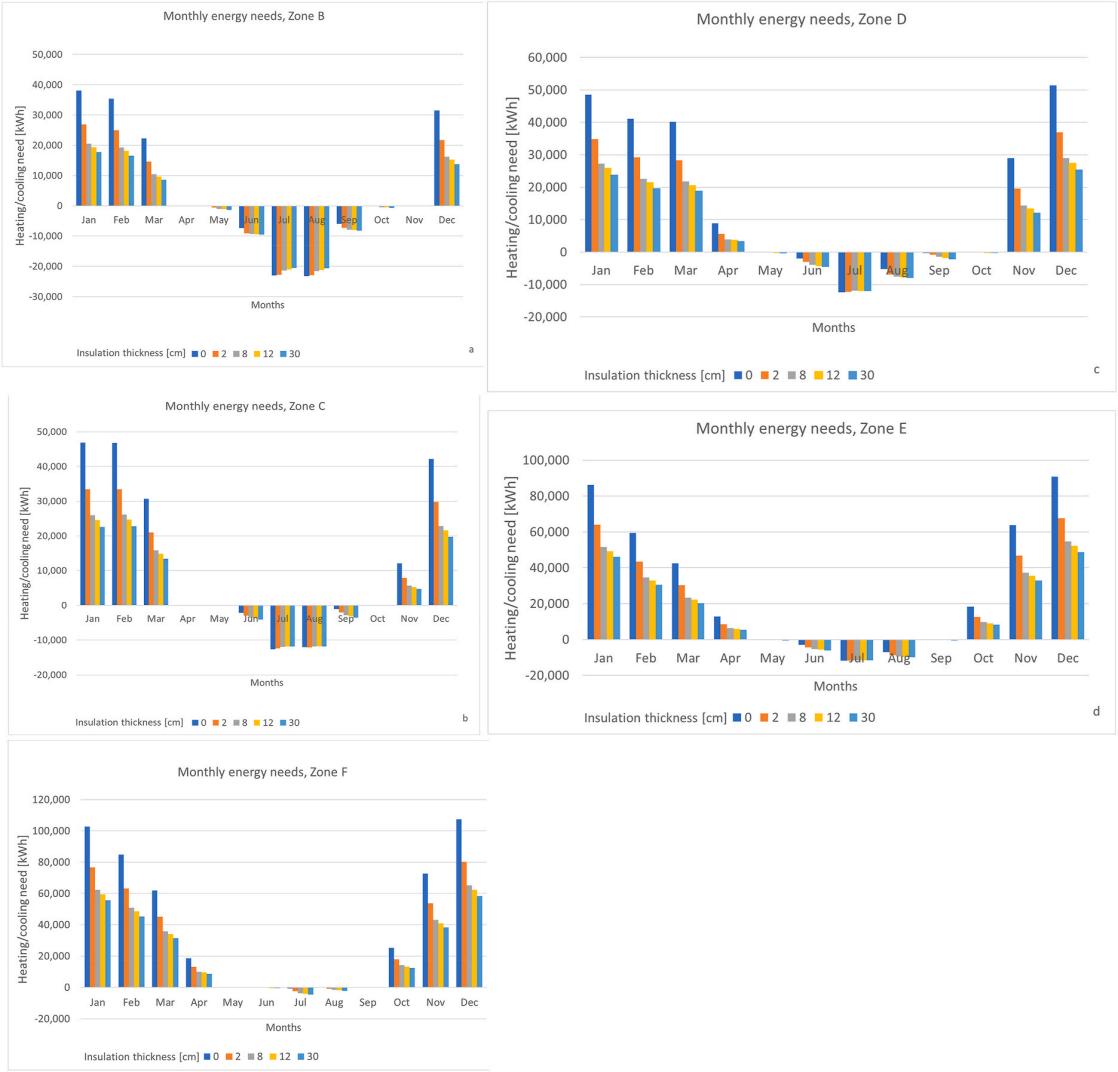
Monthly energy need (heating – positive values, cooling – negative values) for an apartment building, varying the thickness of the insulator, at different climatic zones (a – climatic zone B, b – climatic zone C, c – climatic zone D, d – climatic zone E, e − climatic zone F).
